# Experimental and Electrochemical Research of an Efficient Corrosion and Scale Inhibitor

**DOI:** 10.3390/ma12111821

**Published:** 2019-06-05

**Authors:** Yanmin Chen, Weiwei Xing, Ling Wang, Lingxia Chen

**Affiliations:** 1 College of Chemistry and Chemical Engineering, Zhengzhou Normal University, No.6 Yingcai Street, Zhengzhou 450044, China; wanglingzznu@163.com (L.W.); clingxia@vip.163.com (L.C.); 2 Faculty of General Education, Zhengzhou Technology and Business University, No.8 Qiancheng Road, Zhengzhou 451400, China; xing19840308@163.com

**Keywords:** carbon steel, corrosion inhibition, electrochemistry, morphology analysis, scale inhibition

## Abstract

A novel corrosion and scale inhibitor (TPP) containing tobacco stem extract (TSE), polyepoxysuccinic acid (PESA), and polyaspartic acid (PASP) was obtained by the optimal proportion of the orthogonal test. The anticorrosion effect of TPP for carbon steel was researched by static weight-loss method, on-line simulated dynamic test, electrochemical measurement, and scanning electron microscopy (SEM). The results showed that TPP could protect carbon steel efficiently with a maximal corrosion inhibition rate of 85.7% and it was a mixed-type corrosion inhibitor, mainly exhibiting cathode suppression capacity. Simultaneously, the results of calcium carbonate deposition experiment indicated that the scale inhibition rate of TPP was up to 100%.

## 1. Introduction

Organic phosphorous compounds, such as 1-hydroxy ethylidene-1,1-diphosphonic acid (HEDP) and amino trimethylene phosphonic acid (ATMP), which possess the dual effects of scale inhibition and corrosion resistance, greatly promoted the development of water treatment technology [1,2,3,4]. Based on the advantages of chemical stability, resisting hydrolysis due to the carbon-phosphorus (C–P) bond in their structures, obvious threshold effect (maximum inhibition rate at a certain concentration), and good synergistic effect, a large number of composite agents were prepared and applied in various circulating water systems [5,6,7]. Unsatisfactorily, these phosphoric compounds usually caused eutrophication and oxygen deficiency in the water, which led to the death of aquatic organisms.

In the 1980s, a series of low-phosphorus scale and corrosion inhibitors, such as 2-phosphonylbutylalkane-1,2,4-tricarboxylic acid (PBTCA) and dimethyl-phosphonyl-amino-ethanesulfonic acid (DMPAES), were developed to reduce pollution of the phosphorus emission to environment [8,9]. The molecular structure of PBTCA contained both phosphonic acid groups (–PO_3_H_2_) and carboxylic acid groups (–COOH), and DMPAES contained sulfonic acid (–SO_3_H) and –COOH groups. The existence of multifunctional groups endowed them with stronger performance capacities for corrosion mitigation to steel, compared to the previously used organic phosphonic acid. In order to further reduce the content of phosphine, a number of polymeric scale and corrosion inhibitors were synthesized by copolymerizing phosphorus-carboxylic acid polymers (such as inorganic monomer subphosphate) with organic monomer (such as acrylic acid (AA) and maleic acid (MA)) [10,11,12]. 

**Figure 1:**
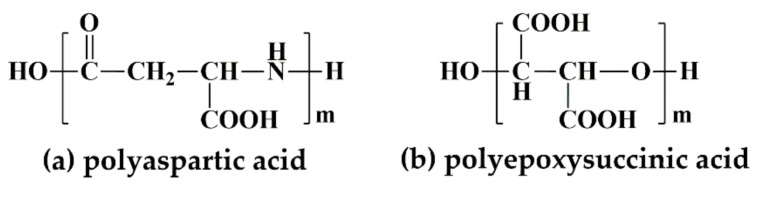
Molecular structures of (**a**) polyaspartic acid and (**b**) polyepoxysuccinic acid.

With the growing concern about environmental protection, novel environment-friendly inhibitors had been developed, for instance, biodegradable polyaspartic acid (PASP), polyepoxysuccinic acid (PESA), and S-carboxyethyl thiosuccinic acid (CETSA) [13,14,15,16]. PASP with carboxyl groups in the side chains (shown in Figure 1a) possessed chelation and dispersion properties, and was more easily biodegradable than PAA, due to nitrogen atoms inserted into the main chain. At present, PASP, as a multifunctional polymer material, which has replaced PAA and its copolymers in many fields, has gained widespread scholarly interest. Another phosphorus-free and nitrogen-free inhibitor, PESA, exhibiting good synergistic effect, was suitable for high alkali and high hardness water systems, attributing to the stability of pentatomic chelates, which are formed by metal ions and oxygen atoms in polymer chains (Figure 1b). CETSA, containing three carboxyl groups and one sulfonic acid group, had wide adaptation pH (5.5–9.0), efficient heavy metal capture capability, water retention and dispersion, and it can be chelated with various metal ions to effectively inhibit scale and metal corrosion by the dense protective coating on the metal surface [17]. Despite these excellent properties, their high cost severely limited their large-scale industrial applications. Thus, it is desirable and urgent to prepare low-cost composite water treatment agents through the synergistic effect between components [18,19,20,21].

Furthermore, the plant extracts of *Morus alba* pendula [22], ginkgo [23], *Musa paradisiaca* [24], *Gum arabic* [25], *Sida cordifolia* [26], garlic peel [27], and olive [28] have been widely studied and examined as effective inhibitors in recent years. However, the inhibition behavior of tobacco stem extract (TSE) has rarely been reported [29]. The chemical composition of TSE largely consists of reducing sugar (such as, glucose and fructose), organic acids (mainly malic acid, oxalic acid, and citric acid) and amino acid (for example, proline and asparagine). These chemical constituents would have anti-corrosion and scale inhibition effects due to their abundant functional groups, such as –COOH and –OH, and the oxygen atoms are regarded as effective adsorption centers [30].

In this work, the anti-corrosion and scaling ability of TSE were firstly studied. Then, TSE was compounded with PESA and PASP to construct a composite inhibitor of TSE/PASP/PESA (TPP) with improved corrosion and scale inhibition, and the composition of TPP was optimized via the orthogonal test. Finally, the corrosion and scale inhibition efficiencies of TPP were investigated by weight-loss experiment, scanning electron microscopy (SEM), static deposition method, and on-line simulated dynamic test. The corrosion resistance behaviors of TPP for carbon steel were further researched by potentiodynamic polarization and electrochemical impedance spectroscopy (EIS). 

## 2. Experiment 

### 2.1. Materials

Tobacco stem extract (TSE) was prepared by extraction of 20 g of tobacco stem in 400 mL × 2 of distilled water at 50 °C for 3 h with a gentle stirring. The TSE was concentrated to a concentration of ca 20 wt %. Polyepoxysuccinic acid (PESA) and polyaspartic acid (PASP) were purchased from Shandong Yousuo Chemical Technology Inc. (Linyi, China). A3 carbon steel specimens (50 mm × 25 mm × 2 mm), containing 0.19% carbon, 0.52% manganese, 0.28% silicon, 0.022% sulfur, and 0.018% phosphorus, from Hebei Legend Water Treatment Inc. (Shijiazhuang, China), were polished with different sandpapers (400, 800, 1200) and then cleaned ultrasonically with distilled water and ethanol respectively. Other reagents were commercially available chemical reagents and used as received.

### 2.2. Corrosion Inhibition Performance

#### 2.2.1. Weight Loss Measurements and Morphology Characterization

A3 carbon steel test pieces of known weight (precisely up to 0.0001 g) were immersed in a beaker containing 500 mL of tap water with and without inhibitors for 72 h at 60 °C. Then, the A3 carbon steel test pieces were washed by 10 g/L hexamethylenetetramine solution in 3 mol·L^−1^ HCl, distilled water, 60 g/L NaOH solution, distilled water, ethanol, respectively. The A3 pieces were dried and weighted. The corrosion rate and the anti-corrosion efficiency of the inhibitor were calculated according to Equations (1) and (2), respectively [31].

(1) $$X=\frac{87600\times \Delta m}{t\cdot A\cdot \rho },$$

where *X*, Δ*m*, *t*, *A*, and *ρ* are the corrosion rate (mm/a), weight loss (g), immersion time (h), test sample area (cm^2^), and density (g/cm^3^), respectively. The inhibition efficiency (*η*_1_) of the inhibitor was calculated by Equation (2):

(2) $$\eta_{1}(\%)=(1-\frac{X}{X_{0}})\times 100,$$

where *X* and *X*_0_ are the corrosion rates calculated from the weight loss with and without the inhibitor.

The morphology of the A3 piece was observed with a JMS-6510 scanning electron microscopy (SEM) (JEOL, Tokyo, Japan). The samples were mounted directly onto the SEM sample holder using double-sided sticking tape prior to the measurements.

#### 2.2.2. Electrochemical Measurements

The electrochemical measurements were carried out on a CS2350 electrochemical workstation (Corrtest Instrument, Wuhan, China) in test solutions with and without the inhibitor through a typical three-electrode system. This electrode system consisted of a working electrode (A3 carbon steel specimen), auxiliary electrode (platinum electrode), and reference electrode (saturated calomel electrode). At first, carbon steel was at open circuit potential (OCP) for 0.5 h to achieve a stable state. Then, the electrochemical impedance spectroscopy (EIS) experiments were performed with the initial voltage of *E*_OCP_, at the frequency range of 0.01–10^5^ Hz, the alternating current excitation signal amplitude of 10 mV and the quiet time of 2 s. Finally, the potentiodynamic polarization curves were swept from 250 mV to + 250 mV at a scan rate of 0.5 mV∙s^−1^. All tests of the same conditions were performed three times to obtain good reproducible results.

### 2.3. Scale Inhibition Performance

To 250 mL distilled water, 20.0 mL of CaCl_2_ solution (*c* = 16.7 g/L), a certain amount of inhibitor, 20.0 mL of borax buffer (pH = 9.0), and 20.0 mL of NaHCO_3_ solution (*c* = 25.2 g/L) were added in turn. The solution was diluted to 500.0 mL, where the Ca^2+^ concentration was about 240 mg/L. 

Then, the solution was transferred to a conical flask for the deposition experiment. The solution was thermostated at 60, 70, and 80 °C for 10 h, respectively. The concentration of Ca^2+^ was determined by disodium ethylenediamine tetraacetate (EDTA) titration method and the scale inhibition rate (*η*_2_) was calculated by Equation (3) [32]:

(3) $$\eta_{2}(\%)=(\frac{C_{1}-C_{2}}{C_{3}-C_{2}})\times 100,$$

where *C*_1_ and *C*_2_ indicates the Ca^2+^ concentrations with and without the inhibitor after deposition experiment, and *C*_3_ is the Ca^2+^ concentration before the deposition experiment.

### 2.4. Dynamic Simulation Test

Dynamic simulation test was performed on a NJHL-C industrial circulating water dynamic simulation device (Nanjing Technology University, Nanjing, China). The volume of dynamic simulation circulating water system, circulation volume flow, concentration factor, test time, and inlet and outlet temperature were 100 L, 180 L/h, 2.5, 360 h, (32 ± 0.2) °C, and 38 °C, respectively. Appropriate dosage of the inhibitor calculated from the product of optimum concentration and system volume was put into the circulating system at the beginning of the test, and then added every other day based on the amount of supplementary water [31].

## 3. Results and Discussion

### 3.1. Weight-Loss Experiment

#### 3.1.1. Corrosion Inhibition of TSE, PESA, and PASP

**Figure 2:**
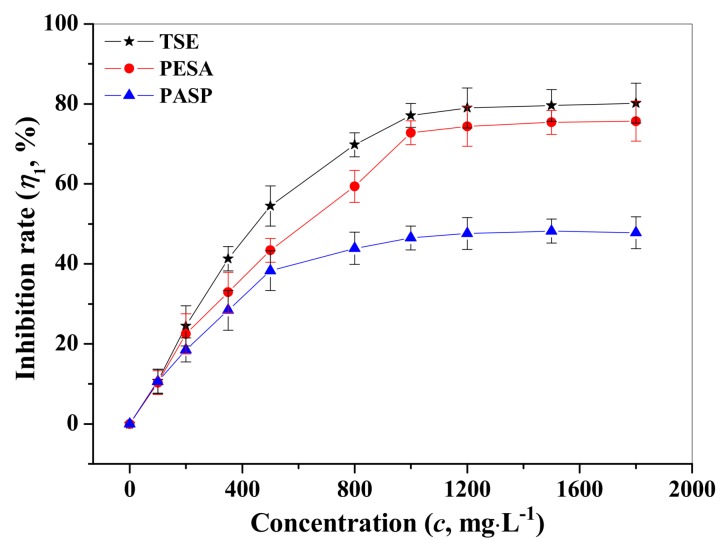
Changes of the corrosion inhibition rates of tobacco stem extract (TSE), polyepoxysuccinic acid (PESA), and polyaspartic acid (PASP) with increasing concentration from 0 to 1800 mg/L at 60 °C.

The effects of TSE, PESA and PASP concentrations on the protection ability of carbon steel were represented in the form of concentration vs. inhibition efficiency (Figure 2). 

As shown in Figure 2 and Table 1, the inhibition rates increased with the increasing concentration of TSE, PESA, and PASP, and reached 80.2% (TSE), 75.7% (PESA), and 47.8% (PASP) at 1800 mg/L. The improvement of the inhibition performance attributed to more coverage of the metal surface because of the adsorption of inhibitor molecules onto the carbon steel surface, which finally reduced the corrosion rate. These results indicated that the molecular structures of TSE, PESA, and PASP played an important role in the inhibition efficiency. In this work, three inhibitors all had non-bonding electron on the oxygen and nitrogen atoms, which helped them to adsorb on the carbon steel surface.

The number of electron-donating functional groups could also affect the adsorption tendency of the inhibitor, i.e., with more electron donating functional groups, the adoption would be stronger and the inhibition efficiency would be higher. Thus, TSE containing the most adsorption centers of N, S, O possessed the highest protection ability [19], followed by PESA containing two −COOH groups in its monomer. PASP was the least efficient inhibitor resulted from the only one −COOH group in its monomer.

#### 3.1.2. Corrosion Inhibition of Ternary Complex TPP

The corrosion inhibition efficiency order was TSE > PESA > PASP. Considering the good synergistic effect of PESA and PASP, a series of ternary compound formula, which potentially improved the inhibition performance, were obtained through the orthogonal design L_9_(3^3^) experiment (shown in Table 2). In addition, the corrosion inhibition rates of TSE, PESA, and PASP increased slightly upon the concentration from 1000 to 1800 mg/L, compared with the significant increase from 0 to 1000 mg/L. Thus, the anti-corrosion effects of composite formula were investigated at the concentration of 1000 mg/L to contrast with single component TSE, PESA, and PASP. As shown in Table 2, the corrosion inhibition rates of the nine composite formula were all above 79%, which was greater than 77.1% of TSE, 72.8% of PESA and 46.5% of PASP, and the proportion of *c*_TSE_/*c*_PESA_/*c*_PASP_ = 3:3:2 (No.9) was the optimum formula (TPP) with the corrosion inhibition rate of 87.2%. Furthermore, according to the range (*R*) analysis (seen in Table 3), it could be inferred that TSE was the main factor affecting the corrosion resistance, followed by PESA, and finally PASP. The order of influencing factors was consistent with that of inhibition rate of single agent for carbon steel.

**Figure 3:**
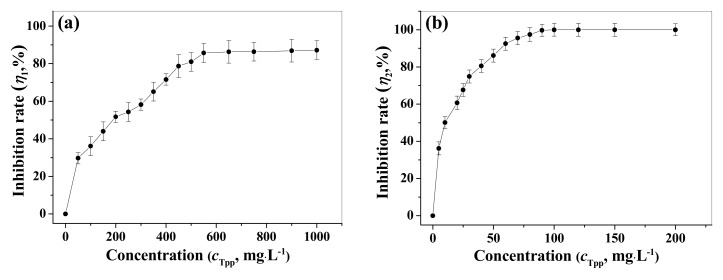
Changes of the corrosion (**a**) and scale (**b**) inhibition rates of TPP during the increasing concentration from 50 to 1000 mg/L and from 5 to 200 mg/L at 60 °C.

As shown in Figure 3a, the corrosion inhibition performance of ternary complex TPP (No.9) gradually improved as the concentration increased from 50 to 550 mg/L and maintained stability at *c*_TPP_ > 550 mg/L. Thus, the optimal concentration was 550 mg/L with the corrosion inhibition rate of 85.7%.

### 3.2. Morphology Analysis

**Figure 4:**
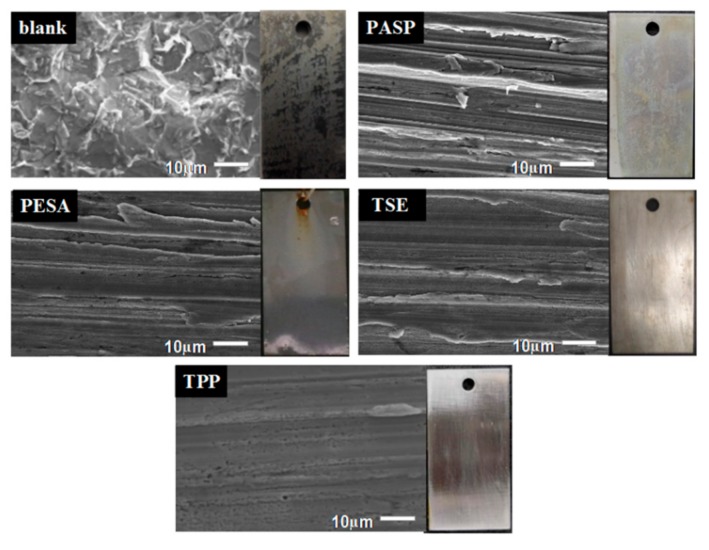
SEM morphologies and optical images of the carbon steel specimen immersed in blank, PASP, PESA, TSE, and composite inhibitor TPP at the concentration of 1000 mg/L.

The optical images of carbon steel specimens after the weight-loss experiment are shown in Figure 4. Clearly, the specimen in the absence of the agent was severely corroded with a very rough and deep gully surface, and the corrosion phenomenon was variously relived after adding PASP, PESA, TES, or TPP. In the presence of the composite inhibitor TPP, the specimen presented a smooth and flat surface, while the other three specimens had different degrees of pitting and surface erosion, among which the that of the specimen taken from PASP solution was more serious than that of TSE and PESA. These results corresponded to the data of weightlessness tests.

The corrosion behavior of carbon steel was further obtained through scanning electron microscope (SEM). SEM morphologies of the carbon steel specimens from blank, PASP, PESA, TSE, and TPP solution were observed at 20 kV accelerating voltage and 2000× magnification (Figure 4). As shown in Figure 4, the specimen in composite inhibitor TPP solution had a complete and smooth surface, the specimens in TSE, PASP, and PESA solution were corroded to various degrees, while the blank specimen was seriously eroded and its surface had much pitting and gaps. These results illustrated that the ternary composite agent had better corrosion inhibition performance than one-component PASP, PESA, and TSE at the same dosage, which attributed to the good synergistic effects between components, and it was also consistent with the above corrosion inhibition rates and optical images.

### 3.3. Electrochemical Analysis

#### 3.3.1. Potentiodynamic Polarization Curves

**Figure 5:**
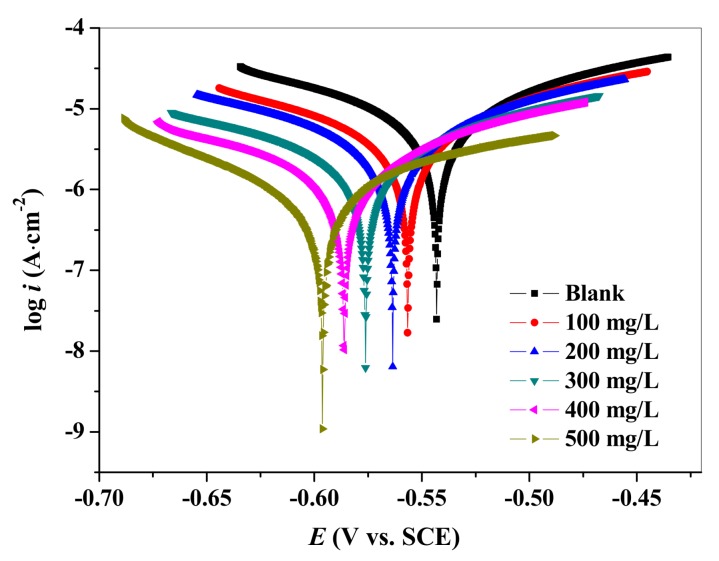
Polarization curves for carbon steel in the absence and presence of different concentrations of TPP at 25 °C.

The potentiodynamic polarization curves (Tafel) of carbon steel in the solutions without and with different concentrations of TPP were represented in Figure 5. 

It can be seen from Figure 5 that the corrosion-current density values gradually shifted from 1.8 × 10^−7^ A·cm^−2^ (Blank) to 2.2 × 10^−8^ A·cm^−2^ (*c*_TPP_ = 500 mg/L), and this indicated that the carbon steel corrosion was effectively inhibited. Furthermore, upon the increasing concentration of TPP, the corrosion potential of carbon steel moved to a negative direction with all variation values lower than 85 mV. These phenomena demonstrated that TPP was a mixed type inhibitor, which not only inhibited the anodic metal dissolution reaction, but also controlled the cathodic oxygen absorption reaction [33,34]. The corrosion inhibition ability was improved with the addition of the TPP concentration, which can be explained by the fact that more TPP molecules were absorbed on carbon steel surface, occupying the active sites to protect the carbon steel. In order to show clearly the mechanism of the corrosion inhibitor, cathodic polarization and anodic polarization were discussed separately. Compared with those of the blank solution, the tendency of the cathode branch corrosion current density to decrease was significantly faster than that of the anode branch. This revealed that the inhibition of the cathode was significantly larger than that of the anode. Therefore, it can be judged that TPP was a mixed-type corrosion inhibitor which mainly inhibited the cathode reaction [33].

The composite inhibitor TPP could protect carbon steel from corrosion, as attributed to the following two reasons: 

(1) The corrosion of carbon steel in neutral-alkaline aqueous solution was significantly exacerbated by hydroxyl ions, which formed intermediate catalytic complexes on the steel surface. The anodic corrosion reaction was as follows [34]:

$$\begin{array}{l} \mathrm{Fe}+{\mathrm{OH}}^{-}↔\mathrm{Fe}{\left(\mathrm{OH}\right)}_{\mathrm{ads}}+e^{-}\\ \mathrm{Fe}{\left(\mathrm{OH}\right)}_{\mathrm{ads}}\to {\mathrm{FeOH}}^{+}+e^{-}\\ {\mathrm{FeOHH}}^{+}+H^{+}↔{\mathrm{Fe}}^{2+}+H_{2}O \end{array}$$

Then, the electrons flowed to the cathode and were captured by oxygen, the cathodic reaction was as follows:

$$O_{2}+2H_{2}O+4e^{-}\to 4{\mathrm{OH}}^{-}.$$

As shown in polarization curves, the composite inhibitor TPP could suppress the cathodic reaction. In the presence of TPP, the reduction of cathode oxygen was inhibited, and then the dissolution of anode metal was also prevented.

(2) The corrosion inhibitor molecule was more easily combined with iron ions to form a coordination bond, which effectively isolated the medium solution from the iron substrate, thereby achieving a good corrosion inhibition effect.

#### 3.3.2. Electrochemical Impedance Spectroscopy (EIS)

**Figure 6:**
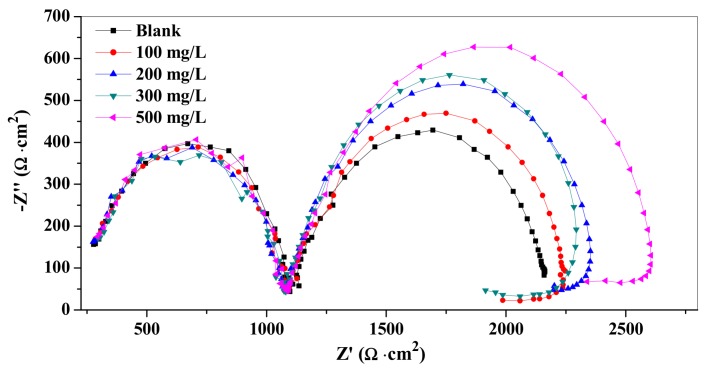
Nyquist plots for carbon steel in the absence and presence of different concentrations of TPP at 25 °C.

The capacitive and inductive behaviors of carbon steel were studied by an electrochemical impedance experiment. The Nyquist plots of the metal without and with different concentrations of TPP are shown in Figure 6. According to Figure 6, the impedance response of carbon steel significantly changed after the addition of TPP compared with the blank. This phenomenon is attributed to an increase in substrate impedance with the increment of inhibitor concentrations. The Nyquist diagram of the blank sample and the added corrosion inhibitor appear as two capacitive reactance arcs with a semicircular shape, one capacitive loop at the high frequency area (HF) due to a finite diffusion impedance of carbon steel electrode, and the other capacitive loop at the intermediate frequency area (MF) related to the electric double layer capacitance and charge-transfer resistance. Moreover, this inductive behavior was observed at low frequencies (LF) in the presence of TPP, which attributed to the relaxation process of the adsorption of inhibitor molecules on the carbon steel surface [35].

In addition, the radius of the capacitive arc in the MF region increased upon the raising of inhibitor concentration, indicating that the corrosion inhibitor could be adsorbed on the surface of carbon steel and form a protective film to improve the corrosion resistance of carbon steel, thereby reducing the corrosion rate.

### 3.4. Deposition Experiment

#### 3.4.1. Scale Inhibition of TSE

**Figure 7:**
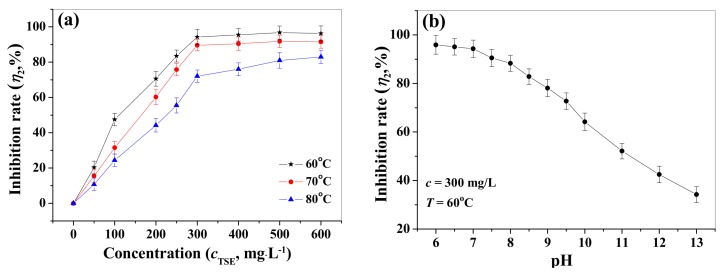
The effects of temperature (**a**) and pH (**b**) on the scale inhibition rates of TSE.

Similar to the corrosion resistance, the inhibition effect of TSE concentration on the calcium carbonate scale gradually improved and stabilized at the concentration of 300 mg/L with the inhibitor rate of 94.3% (60 °C). The anti-fouling tendencies of TSE at 70 °C and 80 °C were the same as that of 60 °C (shown in Figure 7a). However, the scale inhibition rates at 60 °C were larger than that of 70 °C, followed by 80 °C, which was attributed to the decreasing adsorption rate at higher temperature caused by the exothermic adsorption reaction. Furthermore, the effect of pH on the scale inhibition performance of TSE (300 mg/L, 60 °C) was investigated, and the results showed that the inhibition rates decreased from 96% to 34% upon increasing the pH from 6.0 to 13.0 (Figure 7b). This was ascribed to the reduction in the content of effective inhibition component in the alkaline environment, which was due to the neutralization reaction. On the other hand, Ca^2+^ ions easily formed scale in strong basic media, thus weakened the scale-resistance efficiency of TSE. In spite of this, the inhibition rate of TSE still reached to 64% at pH = 10.0. These results indicated that TSE was not only applicable to acidic/neutral, but also available to weakly alkaline environments.

#### 3.4.2. Scale Inhibition of Ternary Complex TPP

The scale inhibition effects of composite formula consisting of TSE, PESA, and PASP were further evaluated and the results were shown in Table 1. It was clearly shown that the inhibition rates of nine composite formula were all larger than 94.3% of TSE under the same concentration of 300 mg/L, and the proportion of *c*_TSE_/*c*_PESA_/*c*_PASP_ = 3:3:2 (TPP) displayed the best anti-fouling efficiency with the inhibition rate of 100%. These results were accordant with the data in Table 1 regarding corrosion inhibition performance. Also, the *R* values shown in Table 2 demonstrated that the order of influencing factors was TSE > PESA = PASP, which revealed that TSE played an important role in the ternary complexes.

Furthermore, as shown in Figure 3b, the anti-scaling effect of the composite inhibitor TPP gradually improved upon the increasing of TPP concentration and reached 100% at *c*_TPP_ = 100 mg/L, then stabilized. Thus, TPP possessed excellent scale inhibition property and almost completely inhibited the deposition of calcium carbonate scale with the dosage of 100 mg/L. The scale inhibition behavior attributed to the strong chelation of composite inhibitor with Ca^2+^, which decreased the free Ca^2+^ concentration, thereby reducing the combination odds of carbonate ions with calcium ions. In addition, N and O atoms of composite inhibitor TPP, by means of lone pair electrons, were irreversibly adsorbed on the main growth site of the CaCO_3_ crystal, thus leading to lattice distortion and reduction in the growth rate of CaCO_3_ scale [36,37].

### 3.5. Dynamic Simulation Test

In order to further investigate the inhibiting effect of the composite corrosion and scale inhibitor in a circulating cooling water system, a dynamic simulation test was carried out. Firstly, the amount of 99 g inhibitor, which was obtained from the product of system volume (180 L) and optimum concentration (550 mg/L), was put into the simulated system. Then, according to the amount of make-up water, the 550 mg/L dosage was added into a dynamic system every other day in the test period (15 d). The results showed that the corrosion rate of carbon steel tube, dirt deposition rate, and limit fouling resistance in the presence of TPP were 0.043 mm·a^−1^, 12.58 mg·cm^−2^·m^−1^ and 0.51 × 10^−4^ m^2^·K·W^−1^, respectively, less than the upper limit values of 0.075 mm·a^−1^, 15 mg·cm^−2^·m^−1^, and 0.86 × 10^−4^ m^2^·K·W^−1^ [38]. The above experiment data indicated that the composite inhibitor could both effectively delay corrosion of carbon steel equipment and pipelines and prevent fouling deposits.

## 4. Conclusions

A composite water treatment agent of TSE/PASP/PESA (TPP) was developed as a corrosion and scale inhibitor in a recirculating cooling water system. TPP could not only inhibit the corrosion for A3 carbon steel but also had excellent inhibition efficiency for calcium carbonate scale. The corrosion inhibition efficiency of the TPP for carbon steel gradually improved with the increasing of TPP and reached a maximal inhibition rate of 85.7% at the concentration of 550 mg/L. The electrochemical polarization curve and EIS analysis results showed that TPP could suppress the cathode and thus exhibited a high corrosion inhibition efficiency. A static scale deposition test indicated that TPP could achieve an excellent calcium carbonate scale inhibition rate of near 100% at the concentration of 100 mg/L.

## Figures and Tables

**Table 1:** The corrosion inhibition rates (*η*_1_) of TSE, PESA, and PASP for A3 carbon steel upon increasing the concentration from 0 to1800 mg/L (60 °C).

Concentration (mg/L)	*η*_1,TSE_ (%)	*η*_1,PESA_ (%)	*η*_1,PASP_ (%)
100	10.7	10.3	10.6
200	24.5	22.5	18.5
350	41.3	32.9	28.4
500	54.5	43.4	38.3
800	69.8	59.4	43.9
1000	77.1	72.8	46.5
1200	79.0	74.4	47.6
1500	79.6	75.4	48.2
1800	80.2	75.7	47.8

**Table 2:** The corrosion (*η*_1_) and scale (*η*_2_) inhibition rates of ternary compound formula (60 °C).

No.	*c*_TSE_/*c*_PESA_/*c*_PASP_	*η*_1_ (%) *c*_total_ = 1000 mg/L	*η*_2_ (%) *c*_total_ = 300 mg/L
1	1:1:1	81.6	97.6
2	1:2:2	80.9	97.3
3	1:3:3	79.2	97.1
4	2:1:2	81.9	97.8
5	2:2:3	82.4	98.2
6	2:3:1	83.6	98.6
7	3:1:3	84.1	99.0
8	3:2:1	86.4	99.6
9	3:3:2	87.2	100

**Table 3:** The analysis of orthogonal test results.

	*η*_1_			*η*_2_		
	TSE	PESA	PASP	TSE	PESA	PASP
*k*_1_	80.6	82.5	83.9	97.3	98.1	98.6
*k*_2_	82.6	82.9	83.3	98.2	98.4	98.4
*k*_3_	85.9	85.9	81.9	99.5	98.6	98.1
*R*	5.4	3.4	2.0	2.2	0.5	0.5
